# Role of LI-RADS Indeterminate Observations in the Risk of Hepatocellular Carcinoma after HCV Eradication with Direct-Acting Antivirals

**DOI:** 10.3390/diagnostics12051187

**Published:** 2022-05-10

**Authors:** Federica Vernuccio, Roberto Cannella, Giuseppe Cabibbo, Silvia Greco, Ciro Celsa, Francesco Matteini, Paolo Giuffrida, Massimo Midiri, Vito Di Marco, Calogero Cammà, Giuseppe Brancatelli

**Affiliations:** 1 Department of Radiology, University Hospital of Padova, Via Nicolò Giustiniani 2, 35128 Padova, Italy; 2 Section of Radiology, Department of Biomedicine, Neuroscience and Advanced Diagnostics (BiND), University Hospital “Paolo Giaccone”, Via del Vespro 129, 90127 Palermo, Italy; rob.cannella89@gmail.com (R.C.); silviagre90@gmail.com (S.G.); dott.francescomatteini@gmail.com (F.M.); massimo.midiri@unipa.it (M.M.); gbranca@yahoo.com (G.B.); 3 Department of Health Promotion Sciences Maternal and Infant Care, Internal Medicine and Medical Specialties, PROMISE, University of Palermo, 90127 Palermo, Italy; giuseppe.cabibbo78@gmail.com (G.C.); celsaciro@gmail.com (C.C.); paologiuffrida1@gmail.com (P.G.); vito.dimarco@unipa.it (V.D.M.); calogero.camma@unipa.it (C.C.); 4 Department of Surgical, Oncological and Oral Sciences (Di.Chir.On.S.), University of Palermo, 90127 Palermo, Italy

**Keywords:** hepatocellular carcinoma, sustained virologic response, chronic hepatitis C, liver cirrhosis, magnetic resonance imaging

## Abstract

Purpose: To assess whether HCC (LR-5) occurrence may be associated with the presence of Liver Imaging Reporting and Data System (LI-RADS) indeterminate observations in patients with hepatitis C virus infection treated with direct acting antiviral (DAA) therapy. Materials and methods: This retrospective study included patients with HCV-related cirrhosis who achieved sustained virologic response (SVR) after DAA therapy between 2015 and 2019 and submitted to CT/MRI follow-ups with a minimum interval time of six months before and after DAA. Two blinded readers reviewed CT/MRI to categorize observations according to LI-RADS version 2018. Differences in rate of LI-RADS 5 observations (i.e., LR-5) before and after SVR were assessed. Time to LR-5 occurrence and risk factors for HCC after DAAs were evaluated by using Kaplan-Meier method and Cox proportional hazard model, respectively. Results: Our final study population comprised 115 patients (median age 72 years) with a median CT/MRI follow-up of 47 months (IQR 26–77 months). Twenty-nine (25.2%) patients were diagnosed with LR-5 after DAA. The incidence of LR-5 after DAAs was 10.4% (12/115) at one year and 17.4% (20/115) at two years. LR-5 occurrence after DAA was significantly higher in patients with Child Pugh class B (log-rank *p* = 0.048) and with LR-3 or LR-4 observations (log-rank *p* = 0.024). At multivariate analysis, Child-Pugh class B (hazard ratio 2.62, *p* = 0.023) and presence of LR-3 or LR-4 observations (hazard ratio 2.40, *p* = 0.048) were independent risk factors for LR-5 occurrence after DAA therapy. Conclusions: The presence of LR-3 and LR-4 observations significantly increases HCC risk following the eradication of HCV infection.

## 1. Introduction

The incidence of hepatocellular carcinoma (HCC) in at-risk population keeps growing [1] with prognosis depending on tumor stage at diagnosis [2]. In patients with hepatitis C virus (HCV) chronic infection, treatment for hepatitis C came in the early 1980s and has improved over the years with current direct acting antiviral (DAA) therapy achieving viral clearance and then sustained virological response (SVR) rates above 90%, with a good safety profile, and reducing the risk of complications, including hepatic decompensation, bleeding and encephalopathy [3,4,5,6,7,8]. DAA therapy, however, does not eliminate the risk of HCC in presence of cirrhosis [6,9,10,11]. Initial studies reported an unexpected increase of HCC incidence following the eradication of HCV infection with DAAs [5,12,13]. This concern was then rebutted by other studies [6,7,8] and by an individual patient-data meta-analysis [14] that showed either a stable or a decreased overall HCC incidence after DAAs. These controversial data triggered an interesting debate on the residual risk of HCC following the eradication of HCV infection and until now lifelong HCC surveillance is recommended after DAA therapy [11].

CT and MRI diagnosis of HCC are based on several International guidelines and guidance documents [11,15,16,17,18]. Among these, Liver Imaging Reporting and Data System (LI-RADS) [17], which has been integrated into the American Association for the Study of Liver Diseases (AASLD) practice guidance [11], and EASL guidelines [15] are the most used in the Western countries, with the former having a higher sensitivity with similar specificity for HCC diagnosis and allowing for stratification of indeterminate nodules into different risk categories of being HCC [19]. Prior studies analyzing the risk of HCC after DAAs adopted only EASL guidelines for HCC diagnosis, thus potentially skewing the rates of HCC and not considering the role of indeterminate observations that do not meet the imaging criteria for the diagnosis of HCC [5,6,7,8,12,13]. In addition, these studies were unable to identify patients at greater risk for HCC occurrence after SVR. To our knowledge, the rate of HCC before and after SVR by using LI-RADS and the weight of observations at intermediate (LR-3) and high probability for HCC (LR-4) on HCC risk remain unknown. We postulate that the presence of LR-3 and LR-4–i.e., observations with a baseline HCC risk of 33–38%, 64–87% of being HCC, and a risk of 6–15%, 46–48% of progression into HCC, respectively [11,20]–can potentially identify patients at greater risk for HCC occurrence after SVR.

The objective of this study was to assess whether HCC (LR-5) occurrence may be associated with the presence of LI-RADS indeterminate in HCV patients treated with DAA therapy.

## 2. Materials and Methods

This retrospective single-center study was approved by the local Ethical Committee, and a waiver of informed consent was obtained. This Institution is a referral academic center for chronic liver diseases. The authors had control of the data and the information submitted for publication. There was no industry support for this study.

### 2.1. Study Cohort

**Figure 1:**
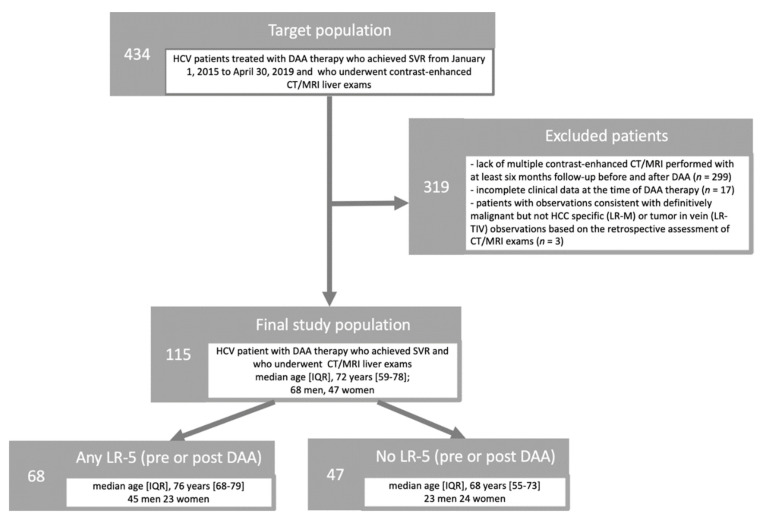
Flowchart of the study populations following the Strengthening the Reporting of Observational Studies in Epidemiology initiative.

Figure 1 portrays the subjects’ accrual flowchart following the Strengthening the Reporting of Observational Studies in Epidemiology (STROBE) initiative [21]. We retrospectively searched the departmental electronic database at our tertiary academic institution for consecutive HCV patients treated with DAA therapy who achieved SVR from 1 January 2015 to 30 April 2019 and who underwent contrast-enhanced CT/MRI liver exams (*n* = 434). SVR was defined as absence of detectable HCV RNA 12 weeks after the completion of treatment [22]. At our Institution, screening and surveillance of patients at risk of HCC are performed based on EASL guidelines and ACR recommendations [15,17].

Patients were considered ineligible for this study in the following cases: (a) lack of multiple contrast-enhanced CT/MRI performed with at least six months follow-up before and after DAA (*n* = 299); (b) incomplete clinical data at the time of DAA therapy (*n* = 17); (c) patients with observations consistent with probably or definitively malignant but not HCC specific (LR-M) or tumor in vein (LR-TIV) observations based on the retrospective assessment of CT/MRI exams (*n* = 3). Indeed, if patients treated with antiviral therapy develop more aggressive forms of HCC, these may appear as LR-M or LR-TIV; these particularly aggressive forms as well as eventual not-HCC malignancy or venous infiltration from malignancy other than HCC may introduce a bias in the analysis of our research question.

### 2.2. Clinical Data

Using the electronic data repository systems we collected patient-related variables, including the patient’s gender, age, body mass index (BMI), diabetes or hypertension, laboratory markers (i.e., alanine transaminase, aspartate transaminase, hemoglobin, International Normalized Ratio, creatinine, bilirubin, platelet count), clinical stratification scores including FIB-4 score [23], Child-Pugh [24,25] and Model for End-Stage Liver Disease (MELD) scores [26], HCV genotype, transient elastography and date of start of DAA therapy.

### 2.3. CT/MRI Technique

Dynamic contrast-enhanced liver CT and MRI exams were performed with a 128-detector row scanner (Somatom Definition AS, Siemens Healthineers, Forchheim, Germany) and two clinical 1.5-T MRI systems (Signa Excite, General Electric, Milwaukee, WI, USA; Achieva, Philips, Amsterdam, The, Netherlands), respectively. The choice of CT vs MRI as well as the use of extracellular or hepatobiliary contrast agents was based on patient clinical evaluation and availability. Specifically, CT is usually performed as it is the most available imaging technique at our center. However, if MRI is available and the patient has no contraindication to MRI or if CT is inconclusive with MRI recommended by the reporting radiologist or by the clinical multidisciplinary team, liver MRI is then performed. Overall, in patients submitted to MRI, hepatobiliary agent is usually preferred if prior recent contrast enhanced CT was performed or in follow-up of cirrhotic patients when liver function is adequate and quality of arterial phase on prior MRI exams was adequate. In the remaining cases, MRI with extracellular agents or is usually performed. CT/MRI protocols and technical parameters were all performed in accordance with the LI-RADS recommendations [27]. CT scan parameters were slice thickness of 2 mm with an interval reconstruction of 2 mm, kV 120, mAs 200 and pitch of 0.6. Liver protocol included precontrast scan, followed by hepatic arterial, portal venous and delayed phases, acquired using the bolus tracking technique. Our liver MRI protocol included in all patients the following sequences: axial T2-weighted sequence usually acquired with both single-shot fast spin-echo (SSFSE or HASTE) technique and with T2-fat saturated technique TSE, axial T1-weighted dual echo spoiled GRE, fat suppressed axial T1-weighted 3D GRE before and after i.e., contrast agent injection and diffusion weighted imaging with *b* values of 0, 150 and 800 sec/mm^2^. On MRI, after the precontrast acquisition, hepatic arterial, portal venous, 3-min phases were acquired with scanning delays after detection of contrast bolus at the origin of the celiac artery of 14, 60, and 180 s, respectively. If extracellular agents or gadobenate dimeglumine were used 5-min equilibrium phase was also acquired. If gadoxetate disodium was used 5-min transitional phase was also acquired. If hepatobiliary agents were used, hepatobiliary phase (HBP) images were obtained 120 and 20 min after intravenous administration of gadobenate dimeglumine and gadoxetate disodium, respectively.

### 2.4. Imaging Analysis

CT/MRI exams at baseline acquitted at least six months before treatment and at all follow-up timepoints after DAA therapy were reviewed to categorize observations based on LI-RADS v2018 algorithm by two readers in consensus (i.e., two trained in liver imaging radiologists) using a clinical picture archiving and communication system (PACS–Impax, Agfa-Gevaert, Belgium) [17]. The readers were blinded to the purpose of the study, patients’ history, date of the DAA therapy, and CT/MRI reports. After excluding observations categorized as noncategorizable (LR-NC), definitely benign (LR-1), probably benign (LR-2), or treated (LR-TR), the readers documented the presence of any observation categorized as at intermediate probability of malignancy (LR-3), probably HCC (LR-4), or definitely HCC (LR-5) at CT/MRI exams before and after DAA therapy.

### 2.5. Statistical Analysis

Statistical analysis was conducted using SPSS Software (Version 20.0. IBM Corp, Armonk, NY, USA) and MedCalc Statistical Software (version 14.8 Ostend, Belgium). Categorical variables were expressed as numbers and percentages and differences were assessed using the Pearson *χ*2 or Fisher exact test. Continuous variables were reported using median and interquartile ranges (IQR) according to the Shapiro-Wilk normality test and the differences were assessed using the Mann-Whitney U test.

First it was tested whether the rate of LR-5 changes before and after DAAs. Incidence of HCC after DAAs was assessed by using the Kaplan–Meier method and the curves were compared by using the log-rank test. Time to LR-5 occurrence after DAAs was calculated as the interval between DAA therapy and post-DAA CT/MRI follow-up exam with diagnosis of LR-5. Then, univariate and multivariate analyses were performed using the Cox proportional hazard models to identify whether clinical and radiological variables could be related to LR-5 occurrence after DAAs. Variables with *p* < 0.1 at univariate analyses were included in the multivariate models. Hazard ratios with 95% confidence intervals (CIs) were calculated at univariate and multivariate analysis. Statistical significance level was set at *p* < 0.05.

## 3. Results

### 3.1. Study Cohort

Our final study population included 115 patients (median age, 72 years) who were deemed eligible for inclusion in the study. Characteristics of the study population are summarized in Table 1. The overall CT/MRI median follow-up time was 47 months (IQR 26–77 months) with a median imaging follow-up of 13 months (IQR 6–38 months) before DAA and 24 months (IQR 12–42 months) after DAA therapy. LR-5 observations were encountered before or after DAA therapy in 68 (59.1%) patients and their presence was significantly higher in patients with older age (median, 76 vs. 68 years), *p* < 0.001), higher FIB-4 score (median, 7.60 vs. 5.46, *p* = 0.034), and with LR-3 or LR-4 observations (*n* = 34, 50.0% vs. *n* = 11, 23.4%; *p* = 0.004). 

### 3.2. Time to LR-5 Occurrence after DAAs

**Figure 2:**
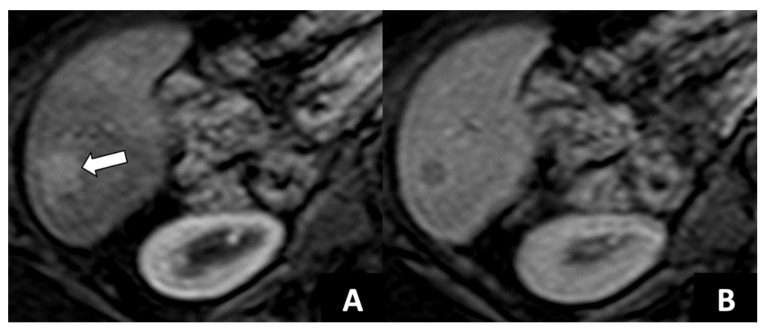
77-year-old woman with history of cirrhosis who achieved sustained virologic response with direct acting antiviral therapy. Contrast-enhanced MRI shows (**A**) a 1.2 cm observation (arrow) with nonrim arterial phase hyperenhancement, and (**B**) nonperipheral washout on portal venous phase, consistent with de novo hepatocellular carcinoma.

**Figure 3:**
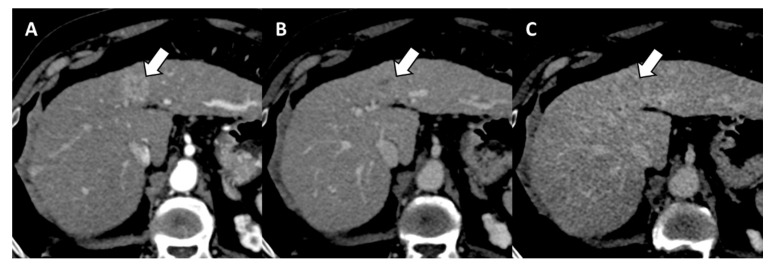
71-year-old woman with cirrhosis who achieved sustained virologic response with direct acting antiviral therapy and history of HCC before HCV eradication. Contrast-enhanced CT reveals a 1.7 cm observation (arrow) nonrim arterial phase hyperenhancement (**A**), and nonperipheral washout (arrows) on portal venous (**B**) and delayed (**C**) phases, consistent with de novo hepatocellular carcinoma at 16 months after DAA.

After DAA therapy, 29 (25.2%) patients were diagnosed with LR-5, including 13 (11.3%) patients with history of pre-DAAs LR-5 and 16 (13.9%) with de-novo LR-5 (Figure 2 and Figure 3). The number of patients diagnosed with LR-5 after DAA therapy was significantly lower compared to those with LR-5 at baseline imaging pre-DAAs (*n* = 29, 25.2% vs. *n* = 52, 45.2%; *p* = 0.001). Patients with LR-5 post-DAAs showed significantly lower levels of albumin (*p* = 0.002), higher Child Pugh score (*p* = 0.010), higher Model for End-Stage Liver Disease (MELD) score (*p* = 0.031), higher liver stiffness (*p* = 0.015), and higher FIB-4 score (*p* = 0.009) as well as a higher frequency of LR-3 or LR-4 observations (*p* = 0.001) (Table 2).

The median time to LR-5 occurrence after DAAs was 16 months (IQR 6–34 months). The cumulative incidence of LR-5 after DAA was 10.4% (12/115) at 1 year, 17.4% (20/115) at 2 years, 20.9 (24/115) at 3 years, and 24.3% (28/115) at 4 years.

**Figure 4:**
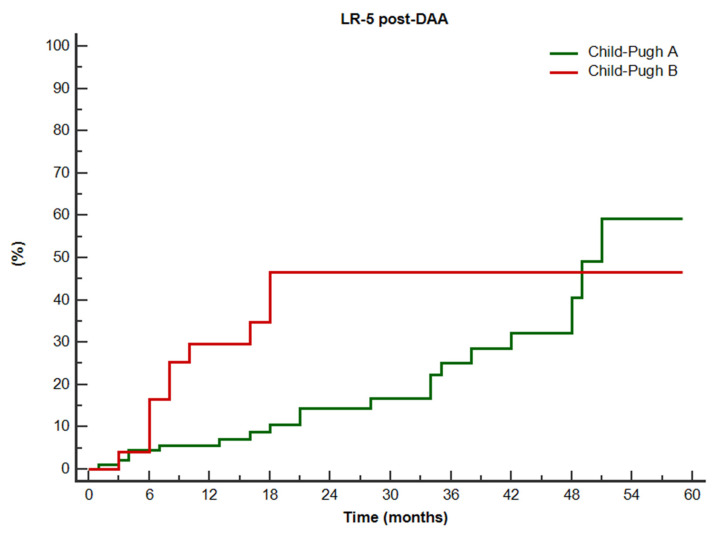
Cumulative Kaplan-Meier curves for LR-5 occurrence after direct acting antivirals, according to the Child-Pugh class. Log-rank *p* for comparison of curves: 0.048.

**Figure 5:**
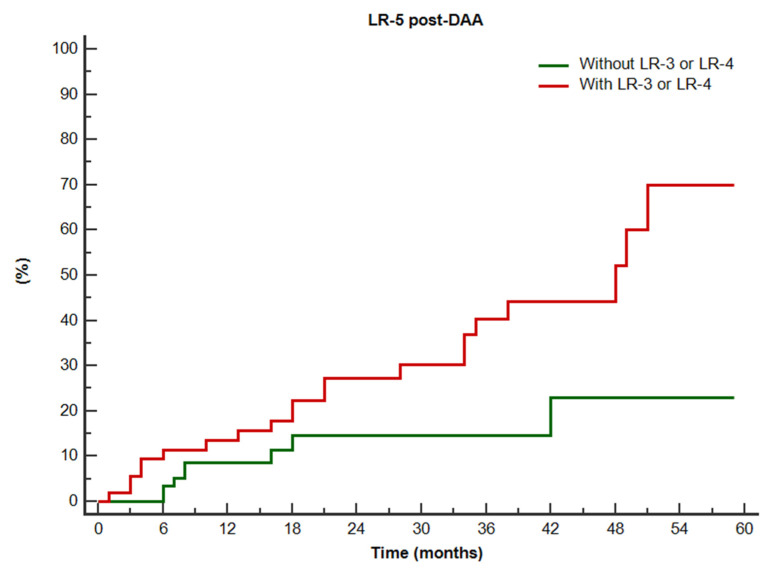
Cumulative Kaplan-Meier curves for LR-5 occurrence after direct acting antivirals, according to the presence of other LR-3 or LR-4 observations. Log-rank *p* for comparison of curves: 0.024.

Kaplan-Meier curves according to Child Pugh class and presence of LR-3 or LR-4 observations are reported in Figure 4 and Figure 5, respectively. Pairwise curves comparisons showed significantly higher cumulative incidence of LR-5 occurrence after DAAs in patients with Child Pugh class B (log-rank *p* = 0.048) and with LR-3 or LR-4 observations (log-rank *p* = 0.024). The cumulative 6-months, 1-year, and 2-years LR-5 incidence ranged from 16.0% to 40.0% in patients in Child Pugh class B (HCC rate: 10/25, 40%) vs. 4.4% to 11.1% in patients in Child Pugh class A (HCC rate: 19/90, 21.1%), and from 11.1% to 24.1% in patients with LR-3 or LR-4 observation (HCC rate: 21/54, 38.9%) vs. from 3.2% to 11.4% in patients without LR-3 and LR-4 (HCC rate: 8/61, 13.1%).

### 3.3. Risk Factors for LR-5 after DAAs

Univariate cox proportional hazards analysis revealed that FIB-4 score (*p* = 0.005) and presence of LR-3 or LR-4 observations (*p* = 0.030) were significant predictors of LR-5 occurrence after DAA (Table 3). Of note, as shown in Table 3, history of LR-5 before DAA therapy (i.e., LR-5 pre-DAA) was not significantly associated with LR-5 occurrence after DAA at univariate analysis (*p* = 0.522). At multivariate analysis, Child-Pugh class B (HR 2.62, 95% CI 1.13–6.02, *p* = 0.023) and presence of LR-3 or LR-4 observations (HR 2.40, 95% CI 1.03–5.74, *p* = 0.048) were independent risk factors for LR-5 occurrence after DAA therapy (Table 3).

## 4. Discussion

In patients with HCV-related cirrhosis, the risk of HCC following HCV eradication with DAA therapy remains high compared to patients who did not receive DAAs [14]. Our results based on imaging follow-up using CT and MRI showed that rate of HCC diagnosed by LI-RADS criteria was lower after HCV eradication in this specific cohort, with an HCC rate of 45% before DAA to an HCC rate of 25% after DAA therapy. This is in accordance with prior studies demonstrating that HCV cure following DAA therapy reduces individual HCC risk [28,29] and with clinical practice update of the American Gastroenterological Association stating that DAA therapy is associated with a reduction in the risk of incident HCC with a similar relative risk reduction in patients with and without cirrhosis [30]. Nonetheless, the rates of HCC reported in our study might seem surprisingly high in comparison with prior studies showing a rate of HCC occurrence of 1–8% and recurrence of 27–29% following DAA therapy [5,6,7,10,13,29]. However, prior studies adopted only EASL guideline for HCC diagnosis, lacked LI-RADS categorization of indeterminate liver observations, and considered separately the risk of HCC occurrence and recurrence. Most importantly, our study is based on radiological assessment of CT and MRI exams in high-risk cirrhotic patients with indeterminate focal liver lesions detected on ultrasound or with prior HCC, thus overestimating the incidence of HCC compared to other studies evaluating non-cirrhotic HCV patients. On the counter side, LI-RADS has a higher sensitivity for HCC diagnosis compared to EASL guidelines due to the analysis of a higher number of imaging features for HCC diagnosis (i.e., enhancing “capsule” as major feature and several ancillary features to adjust the final category) and this may have allowed for identification of a higher number of HCC as compared to prior studies using EASL criteria only [17,19,31].

Another benefit of LI-RADS is the possibility to stratify liver observations based on their estimated probability of being HCC, thus identifying observations at intermediate (LR-3) and high probability (LR-4) for HCC [17,32,33]. According to our multivariate analysis, Child Pugh B and the presence of LR-3 and LR-4 are independent predictors of HCC following HCV eradication. While liver function was previously reported as predictor of HCC risk [5,34,35], to our knowledge the role of LR-3 and LR-4 observations in predicting HCC risk after HCV eradication was not previously investigated. Prior studies identified a higher risk of recurrence after SVR in patients with non-characterized nodules or “imaging dysplastic nodules” at baseline [34,36,37]. Sangiovanni A et al. [38] recently suggested a time-dependent relationship between de novo appearance of HCC after SVR and the presence of radiologically undefined/non-malignant liver nodules. However, the definition of radiologically non-characterized or undefined/non-malignant liver nodules or imaging “dysplastic nodules” is vague and often non-reproducible [39]. When using LI-RADS, it is possible to stratify these observations into different categories reflecting their relative probability of being HCC, which is 33–38% for LR-3 and 64–87% for LR-4 [11,20]. It has been recently shown that DAA therapy does not increase progression of indeterminate observations, including LR-3 and LR-4 [40]. Even though, our results show that patients with LR-3 and LR-4 observations or Child-Pugh B are respectively 2.40 and 2.62 times more likely to have LR-5 observations following HCV eradication, with an HCC incidence at 2-years after DAA therapy of 25.1% and 40.0% in our cohort, respectively. Our results may provide preliminary evidence to justify a close follow-up using CT or MRI after HCV eradication by DAA therapy in HCV patients with LR-3 and LR-4 observations or with more advanced liver disease (i.e., Child-Pugh B) due to the high risk of HCC after DAAs.

Similarly, to prior studies, our results showed a low overall incidence for development of HCC at 1 year and that about one third of LR-5 occur after 2 years from DAA therapy [6,10,35]. Our results demonstrate a median time to LR-5 occurrence after DAAs of 16 months overall but ranged from 8 to 27 months depending on Child-Pugh class and presence of LR-3 or LR-4 observations, with the longest time to LR-5 occurrence being 49 months after HCV eradication. Therefore, regardless of the presence of above-mentioned predictors, we suggest long-term surveillance following HCV eradication, in accordance with current EASL guidelines, AASLD guidance, and AGA practical advices [11,15,30]. In addition, patients at higher risk of HCC after SVR (i.e., those with LR-3 and LR-4) may be potentially benefit of adjuvant therapy after resection/ablation/TACE with checkpoint inhibitors that are drugs being studied by ongoing trials [41,42].

In addition to its retrospective design and the small study cohort, several limitations of our study deserve attention. First, we did not have pathologic proof of LR-3, LR-4, or LR-5 observations, thus potentially skewing our HCC rate or misdiagnosing a small number (≈5%) of LR-5 lesions [11,20]. However, this approach reflects routine practice in guiding clinical decisions for patients at risk of HCC with preferred management being 3–6 months imaging follow-up for LR-3, multidisciplinary discussion for LR-4 to determine individualized management and individualized treatment for LR-5 [43]. Second, we selected only HCV-cured patients that underwent diagnostic CT/MRI for positive US screening, with available long-term follow-ups and with cirrhosis or prior history of HCC in order to comply with LI-RADS requirements for the definition of at-risk patients [17]. Therefore, our results may not be generalizable for HCV-cured subjects with negative US screening. Third, the rate of HCC after DAA therapy included both patients without or with pre-DAA diagnosis of LR-5, thus potentially mixing patients with different baseline risk of HCC. However, LR-5 observations are considered as new lesions for HCC diagnosis according to LI-RADS even in patients with prior HCC and are considered separately from LR-TR viable observations that instead are considered a recurrence of the prior-HCC [17]. Finally, the lack of a control group (i.e., patients with LR-3/4 observations who did not get antiviral treatment, and patients who did not have LR3/4 observations and had no antiviral treatment) does not allow to compare our results with the baseline HCC risk of untreated or DAA not-responsive HCV patients and therefore to establish the exact relationship between the presence of LR3/4 observations, antiviral therapy, and risk of HCC. DAA therapy is currently the standard of care in HCV infection and achieves SVR rates above 90% also in patients with pre-DAA history of HCC.

## 5. Conclusions

To conclude, the presence of observations at intermediate and high probability for HCC increases the risk of HCC following the eradication of HCV infection. The persistence of the risk of HCC after HCV eradication suggests the need for long-term follow-up after DAA therapy, especially in patients with compromised liver function and indeterminate liver observations.

## Figures and Tables

**Table 1:** Characteristics of the final population of patients with any LR-5 diagnosed before or after direct antiviral therapy and in patients without any LR-5 before or after direct antiviral therapy.

Characteristics	Total (*n* = 115)	Any LR-5 (*n* = 68)	Lack of LR-5 (*n* = 47)	*p* Value
**Age** (years)	72 (59.0, 78.0)	76 (68.2, 78.7)	68.0 (55.0, 73.0)	**<0.001**
**Sex**				
Men	68 (59.1)	45 (66.2)	23 (48.9)	0.064
Women	47 (40.9)	23 (33.8)	24 (51.1)	
**HCV genotype**				
1a	4 (3.5)	1 (1.5)	3 (6.4)	0.586
1b	86 (74.8)	51 (75.0)	35 (74.5)	
2	9 (7.8)	6 (8.8)	3 (6.4)	
3	15 (13.0)	9 (13.2)	6 (12.8)	
4	1 (0.9)	1 (1.5)	0 (0)	
**BMI** (kg/m^2^)	24.2 (22.0, 27.6)	25.9 (24.2, 27.6)	25.3 (23.8, 27.3)	0.391
**AST** (IU/L)	64.0 (44.5, 94.7)	69.0 (50.0, 90.0)	59.0 (40.0, 107.0)	0.896
**ALT** (IU/L)	64.5 (41.5, 86.0)	65.0 (49.0, 84.0)	64.0 (37.0, 97.0)	0.871
**Hemoglobin** (g/dL)	13.3 (11.9, 14.6)	13.1 (11.9, 14.7)	13.4 (11.6, 14.5)	0.852
**WBC** (×10^3^/mL)	4900 (3885, 6055)	4810 (4000, 6313)	5050 (3607, 5997)	0.675
**INR**	1.0 (1.0, 1.2)	1.0 (1.0, 1.2)	1.0 (1.0, 1.2)	0.120
**Albumin** (g/dL)	3.6 (3.3, 4.0)	3.6 (3.1, 4.0)	3.7 (3.5, 4.0)	0.204
**Creatinine** (mg/dL)	0.8 (0.7, 1.0)	0.8 (0.7, 1.0)	0.8 (0.6, 0.9)	0.604
**Bilirubin** (mg/dL)	1.0 (0.7, 1.3)	1.0 (0.6, 1.3)	1.0 (0.7, 1.8)	0.352
**Platelet count** (×10^3^/μL)	92.0 (73.0, 138.0)	89.0 (70.0, 128.0)	103.0 (73.0, 145.5)	0.133
**Diabetes**, *n* (%)	27 (23.5)	17 (25.0)	10 (21.3)	0.643
**Hypertension**, *n* (%)	55 (47.8)	34 (61.8)	21 (44.7)	0.575
**TE** (kPa)	13.2 (17.2, 29.6)	20.0 (15.0, 33.8)	16.0 (12.7, 29.0)	0.108
**Child-Pugh Score**	6.0 (5.0, 6.0)	5.0 (5.0, 6.0)	6.0 (5.0, 6.0)	0.979
**Child-Pugh Class**				
A	90 (78.3)	53 (77.9)	37 (78.7)	0.920
B	25 (21.7)	15 (22.1)	10 (21.3)	
**FIB-4 score**	6.45 (3.86, 9.37)	7.60 (4.34, 10.28)	5.46 (2.86, 8.60)	**0.034**
**MELD score**	8.0 (6.0, 10.0)	8.0 (6.0, 10.0)	7.0 (6.0, 10.0)	0.431
**LR-3/LR-4**	45 (39.1)	34 (50.0)	11 (23.4)	**0.004**

Note: Continuous variables are expressed as median and interquartile range (25th to 75th percentile), categorical variables are expressed as numbers and percentages. Statistically significant values (*p* < 0.05) are highlighted in bold. Abbreviations: BMI, body mass index; ALT, alanine transaminase, AST, aspartate transaminase; HCV, hepatitis C virus; TE, transient elastography; MELD, Model for End-Stage Liver Disease; WBC, white blood cells.

**Table 2:** Characteristics of the patients with LR-5 occurrence after direct acting antivirals.

Characteristics	LR-5 Post-DAA (*n* = 29)	Lack of LR-5 Post-DAA (*n* = 86)	*p* Value
**Age** (years)	77.0 (63.0, 78.0)	71.5 (58.0, 72.7)	0.057
**Sex**			
Men	21 (72.4)	47 (57.7)	0.092
Women	8 (27.6)	39 (45.3)	
**HCV genotype**			
1a	0 (0)	4 (4.7)	0.303
1b	22 (75.9)	64 (74.4)	
2	3 (10.3)	6 (7.0)	
3	3 (10.3)	12 (14.0)	
4	1 (1.4)	0 (0)	
**BMI** (kg/m^2^)	25.0 (24.2, 27.6)	25.7 (24.2, 27.6)	0.841
**AST** (IU/L)	73.0 (57.5, 99.5)	70.0 (46.0, 106.0)	0.521
**ALT** (IU/L)	68.0 (49.0, 85.5)	68.0 (50.0, 104.5)	0.747
**Hemoglobin** (g/dL)	13.1 (12.0, 14.4)	13.4 (11.9, 14.5)	0.695
**WBC** (×10^3^/mL)	4130 (3715, 5850)	4950 (3847, 6130)	0.658
**INR**	1.0 (1.0, 1.2)	1.0 (1.0, 1.3)	0.105
**Albumin** (g/dL)	3.5 (3.0, 3.9)	3.7 (3.4, 4.0)	**0.002**
**Creatinine** (mg/dL)	0.8 (0.7, 1.0)	0.8 (0.7, 0.9)	0.448
**Bilirubin** (mg/dL)	1.0 (0.5, 1.6)	1.0 (0.7, 1.3)	0.377
**Platelet count** (×10^3^/μL)	80.0 (62.0, 114.0)	98.0 (70.7, 156.0)	0.063
**Diabetes**, *n* (%)	10 (34.4)	17 (19.8)	0.106
**Hypertension**, *n* (%)	13 (48.8)	42 (48.8)	0.709
**TE** (kPa)	26.0 (16.9, 35.1)	17.0 (13.0, 29.0)	**0.015**
**Child-Pugh Score**	6.0 (5.0, 7.0)	5.0 (5.0, 6.0)	**0.010**
**Child-Pugh Class**			
A	19 (65.5)	71 (82.6)	0.054
B	10 (34.5)	15 (17.4)	
**FIB-4 score**	8.84 (5.46, 11.65)	5.59 (3.25, 8.80)	**0.009**
**MELD score**	8.0 (7.0, 11.0)	8.0 (6.0, 9.0)	**0.031**
**LR-5 pre-DAA**	13 (44.8)	39 (45.3)	0.961
**LR-3/LR-4**	21 (72.4)	33 (38.4)	**0.001**

Note: Continuous variables are expressed as median and interquartile range (25th to 75th percentile), categorical variables are expressed as numbers and percentages. Statistically significant values (*p* < 0.05) are highlighted in bold. Abbreviations: BMI, body mass index; ALT, alanine transaminase, AST, aspartate transaminase; HCV, hepatitis C virus; TE, transient elastography; MELD, Model for End-Stage Liver Disease; WBC, white blood cells.

**Table 3:** Univariate and multivariate cox proportional hazards model for LR-5 occurrence after direct acting antivirals.

	Univariate Analysis			Multivariate Analysis		
Characteristics	Hazard Ratio	95% CI	*p* Value	Hazard Ratio	95% CI	*p* Value
Age	1.03	0.99, 1.07	0.093	1.03	0.99, 1.08	0.093
Male sex	1.52	0.67, 3.46	0.314			
Transient elastography	1.01	0.98, 1.51	0.215			
Child-Pugh Class B	2.13	0.98, 4.62	0.055	2.62	1.13, 6.02	**0.023**
FIB-4 score	1.11	1.03, 1.20	**0.005**	1.07	0.98, 1.16	0.103
MELD score	1.09	0.98, 1.26	0.100			
LR-5 pre-DAA	0.787	0.37, 1.64	0.522			
LR-3 or LR-4 observations	2.47	1.09, 5.61	**0.030**	2.40	1.03, 5.74	**0.048**

Note: Variables with *p* < 0.1 at univariate analyses were included in the multivariate models. Statistically significant values (*p* < 0.05) are highlighted in bold. Abbreviations: 95% CI, 95% confidence interval; DAA, direct-acting antivirals; FIB-4, Fibrosis-4 index for liver fibrosis; MELD, Model for End-Stage Liver Disease.

## Data Availability

Authors will be made available upon reasonable request.
